# Clinical effectiveness of a web-based peer-supported self-management intervention for relatives of people with psychosis or bipolar (REACT): online, observer-blind, randomised controlled superiority trial

**DOI:** 10.1186/s12888-020-02545-9

**Published:** 2020-04-14

**Authors:** Fiona Lobban, Nadia Akers, Duncan Appelbe, Lesley Chapman, Lizzi Collinge, Susanna Dodd, Sue Flowers, Bruce Hollingsworth, Sonia Johnson, Steven H. Jones, Ceu Mateus, Barbara Mezes, Elizabeth Murray, Katerina Panagaki, Naomi Rainford, Heather Robinson, Anna Rosala-Hallas, William Sellwood, Andrew Walker, Paula Williamson

**Affiliations:** 1 Spectrum Centre for Mental Health Research, Division of Health Research, Faculty of Health and Medicine, Lancaster University, Lancaster, UK; 2 Clinical Trials Research Centre, Institute of Child Health, Alder Hey, University of Liverpool, Liverpool, UK; 3 Division of Health Research, Faculty of Health and Medicine, Lancaster University, Lancaster, UK; 4 University College London, Maple House, 149 Tottenham Court Road, London, W1T 7NF UK; 5 e-Health and Primary Care, Primary Care & Population Health Institute of Epidemiology & Health, Faculty of Pop Health Sciences, University College London, London, UK

**Keywords:** Digital health intervention, Relatives, Psychosis, Bipolar, Randomised controlled trial

## Abstract

**Background:**

The Relatives Education And Coping Toolkit (REACT) is an online supported self-management toolkit for relatives of people with psychosis or bipolar designed to improve access to NICE recommended information and emotional support.

**Aims:**

Our aim was to determine clinical and cost-effectiveness of REACT including a Resource Directory (RD), versus RD-only.

**Methods:**

A primarily online, observer-blind randomised controlled trial comparing REACT (including RD) with RD only (registration ISRCTN72019945). Participants were UK relatives aged > = 16, with high distress (assessed using the GHQ-28), and actively help-seeking, individually randomised, and assessed online. Primary outcome was relatives’ distress (GHQ-28) at 24 weeks. Secondary outcomes were wellbeing, support, costs and user feedback.

**Results:**

We recruited 800 relatives (REACT = 399; RD only = 401) with high distress at baseline (GHQ-28 REACT mean 40.3, SD 14.6; RD only mean 40.0, SD 14.0). Median time spent online on REACT was 50.8 min (IQR 12.4–172.1) versus 0.5 min (IQR 0–1.6) on RD only. Retention to primary follow-up (24 weeks) was 75% (REACT *n* = 292 (73.2%); RD-only *n* = 307 (76.6%)). Distress decreased in both groups by 24 weeks, with no significant difference between the two groups (− 1.39, 95% CI -3.60, 0.83, *p* = 0.22). Estimated cost of delivering REACT was £62.27 per person and users reported finding it safe, acceptable and convenient. There were no adverse events or reported side effects.

**Conclusions:**

REACT is an inexpensive, acceptable, and safe way to deliver NICE-recommended support for relatives. However, for highly distressed relatives it is no more effective in reducing distress (GHQ-28) than a comprehensive online resource directory.

**Trial registration:**

ISRCTN72019945 prospectively registered 19/11/2015.

## Background

Relatives and friends of people experiencing psychosis or bipolar provide much unpaid care [1, 2], but at high personal cost emotionally and financially [3–5]. Sample estimates of levels of clinically significant distress and burden in relatives of people with psychosis range from a third [4], to more than 60% of those in early intervention in psychosis (EIP) services [6, 7], with almost half reporting post-traumatic stress symptoms associated with their caring roles [8], particularly linked to episodes of violence, disruptive behaviour and forced admission [9]. Key factors that increase the negative impact of psychosis on carers include: being a female carer [10]; living with the person with psychosis; young patient age and awareness of suicidal ideation [11]; reduced social support and family resources [11, 12]; use of emotion-focused coping strategies [13]; and beliefs that relatives hold about the psychosis, particularly those concerning cause and control [14–16]. Particular challenges for relatives of people with bipolar experiences include high risk of suicide attempts [17], mania related extravagant spending, irritability and disinhibited behaviour [18–20], all of which are associated with feelings of helplessness, anger, and anxiety in their relatives [21, 22]. Historically, the impact of severe mental health problems on relatives has been neglected [23]. However, there is now good evidence that interventions that support relatives can improve both service user [23–25] and carer [26–29] outcomes. The UK Government recognises the need to support carers [30], and the National Institute for Health and Care Excellence (NICE) recommends that all relatives of people with psychosis or bipolar be given carer-focused education and support, and offered structured family intervention to enhance family coping and communication [31, 32]. Despite this, a recent national audit of community mental health services in the UK showed poor implementation, with only 50% of relatives receiving carer-focused education and support and only 12% receiving structured family intervention [33].

Within this context, our aim was to test the clinical and cost effectiveness of an online self-management intervention, based on the principles of psychoeducation and family intervention [34]. The Relatives Education And Coping Toolkit (REACT) was developed with extensive input from relatives and clinicians [35] initially in paper form, supported by staff in EIP services and tested in a feasibility trial which showed a significant reduction in distress for relatives receiving REACT in addition to usual treatment, when compared to those receiving usual treatment only [6]. To increase accessibility, REACT was adapted for this study to be available online and supported by trained relatives with lived experience of supporting someone with a severe mental health problem (REACT Supporters) [36] . REACT included a comprehensive Resource Directory (RD), signposting relatives to other freely available relevant support. We tested REACT including the RD, against the RD only to determine the impact of REACT on relatives’ distress, wellbeing, and support, and to test hypothesised mediators of change including relatives’ beliefs, perceived coping, and amount of use of REACT. We also report the costs of the development and delivery of REACT and the RD, and user experience of REACT. A comprehensive cost effectiveness analysis will be reported elsewhere. A separate study has examined the factors impacting on implementation of REACT in NHS services [37].

## Methods

### Study design

We conducted an online, two-arm, pragmatic, observer-blind, randomised controlled superiority trial open to relatives of people with psychosis or bipolar across the UK. Inclusion criteria were broad and relatives could self-refer into the trial. A nested qualitative study examined user experiences of REACT. Prior to the end of data collection, a trial protocol [38] and a comprehensive statistical analysis plan were published [39, 40]. Reporting follows CONSORT guidance [41].

### Participants

Inclusion criteria were (according to self-report):

- Aged 16 or over
- Living in the UK
- Relative or close friend of someone with psychosis or Bipolar
- Currently experiencing distress (selecting “rather more than usual” or “much more than usual” on GHQ-28 item “Have you recently been feeling nervous and strung up all the time”). This was included to avoid a distress floor effect at baseline (selected item was the most highly correlated most with GHQ total score in the REACT feasibility trial [6].
- Currently seeking help (self-identified)
- Internet access
- Sufficient English fluency to comprehend intervention content

Exclusion criteria were:

- Living in any of six geographical areas by postcode taking part in a parallel implementation study of the same intervention (IMPART) [42].
- Only one relative per service user was allowed to participate, to avoid a clustering effect.

Recruitment took place from 22 April 2016 to 30 September 2017. We used a range of online (Facebook, twitter, charity websites) and offline recruitment strategies (clinical services, third sector providers), all directing potential participants to the study home page, including information about how to take part. At registration, all participants gave online written informed consent, indicated how they had found out about REACT, and provided postal, email and telephone contact details. A convenience sub-sample (*n* = 55) of relatives in the REACT arm who had completed the 24 week follow-up at the time of interviewing were invited to take part in qualitative interviews about their experiences of using REACT, with the aim of recruiting approximately 25 interviewees.

### Randomisation and masking

Eligible participants were randomised using a 1:1 ratio to “REACT (including RD) plus Treatment As Usual (TAU)” or “RD only plus TAU” using web-based variable block randomisation, in which the unit of randomisation was the relative. Participants then received an email indicating which arm of the trial they had been allocated to, and a link to the REACT website, with their username and password. Those in the RD-only arm had access only to the directory pages. All participants were aware that the RD was one component of the REACT intervention, and therefore were likely to have perceived REACT as the “intervention of interest” and the RD as the comparator.

All data were self-reported and predominantly entered online by participants. Data sets submitted by post at follow-up were inputted by the trial manager, blind to allocation. Data were uploaded directly to the Clinical Trials Research Centre (CTRC) database. To prevent bias, the chief investigator, trial manager (TM) and statisticians were blinded to treatment assignment. REACT supporters, clinical supervisors, qualitative interviewer, one CTRC analyst of web usage data and technical staff were unblinded. To minimise unwanted unblinding, all contact with participants was prefaced by a reminder not to disclose trial arm. If the TM was unblinded regarding a particular participant, another blind team member delivered any non-automated reminders and carried out any data entry for that participant.

### Procedures

#### Interventions

##### React

The REACT intervention was built in WordPress, hosted and maintained at Lancaster University, and included: 12 psychoeducation modules; peer support through a moderated group forum; a confidential direct messaging service; and the RD, which pointed relatives to other available resources. The modules addressed important questions relatives have highlighted, and included videos of experts by experience and clinical experts; evidence-based education and strategies; and self-reflection tasks that help users apply the content to their personal circumstances. Module titles were: ‘What is psychosis?’; ‘What is bipolar disorder?’; ‘Managing “positive” symptoms’;;Managing “negative” symptoms’; ‘Managing mood swings’; ‘Dealing with difficult situations’; Managing stress – doing things differently’; ‘Managing stress – thinking differently’; ‘Understanding mental health services’; ‘Treatment options’; ‘Dealing with crisis’; The future and recovery’. A detailed description of each module is presented in the protocol paper, along with screenshots showing the look and feel of the website, [38], and the modules can be freely accessed by visiting reacttoolkit.uk [43].

A “Meet the Team” page informed relatives about who was delivering the content of the site. “Mytoolbox” provided a confidential space for users to save links to information they might find useful in the future including external web links. A blog page offered a flexible space for additional communication with site users, which was editable by the REACT supporters.

REACT Supporters were relatives with lived experience of supporting someone with a mental health problem who were trained to moderate the forum, respond to confidential direct messages from users, and guide users to relevant parts of the toolkit and/or other resources as appropriate. REACT Supporters were hosted by one National Health Service (NHS) mental health trust in England but available to relatives across the UK. They were trained to identify and report risk, and were supervised by two clinical psychologists and an expert relative. A supervision manual, supporter manual and risk protocol were developed for the study and are available on request.

Relatives could access REACT whenever they wished throughout the trial (minimum of 24 weeks to last follow-up for final participant). REACT supporters were available on weekdays 9 am to 4.30 pm excluding public holidays and university closures. Participants were advised to use the intervention as they needed, and emailed reminders (which they could turn off) to visit the website after a week of inactivity.

##### Resource directory (RD)

The resource directory (RD) contained a comprehensive list of national organisations supporting people with psychosis or bipolar and their relatives (such as Rethink, Mind, Carers UK and Bipolar UK), and those for related conditions (such as Anxiety UK and Samaritans (voluntary crisis helpline for people feeling suicidal)). The RD also listed UK government websites offering information and guidance about mental health and related topics, such as NHS Choices, Care Quality Commission, NICE Guidelines and the Department of Work and Pensions, and gave contact details for emergency services, including local NHS mental health services out-of-hours crisis teams. At the end of the study the RD-only participants were given access to the modules, without the forum or direct messaging.

#### Costing the interventions

We assessed all costs relevant to content development for REACT and the RD.

Development costs included: Conception and design of the toolkit; Consultation with service users, relatives and professionals to identify user requirements; Staff time to develop content; Production of videos and images; Design and development of the website; Website infrastructure during development. Delivery costs were computed for a 6-month period (time spent in the trial) and included general infrastructure for hosting the REACT website and the costs of training, supervision, and employment of REACT supporters for 6 months. The costs of developing and delivering the RD were also calculated and half allocated to the intervention arm, and half to the comparator arm.

#### Data collection process

At baseline, participants completed all measures before being randomised. Participants were sent £10 shopping voucher on completion of measures at baseline and 12 week follow up, and £10 or £20, conditional or unconditional on completion at 24 week follow up (secondary randomisation as part of study within a trial SWAT65 https://bit.ly/2WCDMqU).

Partway through the study (8th of September 2017 to 23rd of February 2018), a subgroup of participants were invited to take part in topic guided qualitative interviews (conducted by telephone / video-conferencing) to understand their experiences of using REACT. This sample were randomly selected from a pool of 76 people who had a) been randomized to the intervention arm, b) completed 24 week follow-up at the point of data collection, and c) had consented to be contacted about further research across different levels of use of REACT. All interviews were recorded and transcribed for analysis. A diagram of pathway through the study is given in the protocol [38].

### Outcomes

All outcomes were validated self-report questionnaires collected online using a closed system, presented in order of priority (primary outcome first) at baseline, 12 and 24 week follow-ups. The primary outcome was relatives’ distress at 24 weeks, assessed using the GHQ-28 with Likert scoring (0–3) [44]. Subscales include somatic symptoms, anxiety / insomnia, social dysfunction, and severe depression. Higher score indicates greater distress (score range 0–84).

Secondary outcomes included the relatives’ experience of caring, assessed online at 24-week follow-up using the Carer Wellbeing and Support (CWS) questionnaire [45]; and distress (GHQ-28) and carer experience (CWS) assessed online at 12-week follow-up. CWS provided total wellbeing scores (possible range 0–128), based on levels of concern over the previous 4 weeks about the impact of caring responsibilities on: day-to-day life (e.g. ‘During the past 4 weeks how concerned were you that your caring responsibilities stopped you from having enough time to yourself?’); relationship with the person being cared for; relationship with family and friends; financial situation; physical health; emotional wellbeing; stigma and discrimination; their own safety; the safety of the person they care for. CWS also provided total satisfaction with support from services (possible range 0–51), which assessed how satisfied the relatives were with the information and advice they have received (e.g. ‘In general, how satisfied are you that you have enough information about the condition/illness of the person you care for/support to enable you to feel confident in caring for them/providing support?’); involvement in treatment and care planning; and support from staff. Higher scores indicate better outcome.

Website usage data for each participant was downloaded from the intervention site and summarised for participants in each intervention group.

Participants in the REACT intervention group were asked to rate the following statements at 12 and 24 weeks post-randomisation (based on previously published studies) [46].

- “I always feel supported by the REACT supporters”
- “I always feel supported by the REACT group”
- “I always feel the REACT site was a safe and confidential environment”.

Options for each answer were “strongly disagree”, “disagree”, “agree”, and “strongly agree”.

#### Qualitative interviews

Open questions explored relatives’ general experiences of REACT, factors influencing levels of use, which parts of REACT were used, experience of peer support from REACT and any suggestions for improvements.

#### Safety and adverse events

We assessed the number of low-risk (clear evidence of distress or concerns of risk of harm or abuse towards participants or others, but no immediate or serious threat of severe harm, risk to life or child welfare) and high-risk events (clear evidence of immediate risk to life or child welfare). Risks were identified via online questionnaire red flag items, posts on the REACT forum, direct messages to REACT supporters and by the trial manager during email or telephone participant contact. High-risk events were classed as study adverse events.

### Statistical analysis

Based on data from a feasibility study [6], and in accordance with the rationale detailed in the protocol paper [47], we aimed to recruit 666 relatives of people with psychosis or bipolar to test the primary hypothesis of a mean difference > = 5.0 in GHQ score between arms, assuming a standard deviation of 16.6 units (*p* < 0.05) at 24-weeks follow-up, and with 70% retention.

Mean scores were compared between groups using analysis of covariance (ANCOVA) adjusting for baseline scores and including all participants according to the randomisation scheme. A joint modelling approach was used to assess differences in longitudinal outcomes between the randomised arms adjusted for missingness (at 12-week or 24-week follow-up).

Additional multivariable analyses, using forward stepwise selection and adjusting for baseline GHQ-28 were conducted to identify significant baseline predictors of outcome.

Instrumental variable regression was carried out to estimate the impact of intervention use (number of web page downloads) on outcome. A two-stage least squares estimator (2SLS) was used: the first stage was to fit a model regressing web page downloads on randomisation and the second stage was to regress GHQ-28 at 24 weeks on the fitted values of web page downloads predicted in the previous step. The model was adjusted for baseline GHQ-28 score. All analyses were done using SAS statistical analysis software, version 9.4 and Stata version 14.

### Qualitative analysis

Following the framework approach described by Ritchie and Spencer [48], broad themes were created a priori, based on the research team’s interest in understanding how participants experienced the REACT toolkit, including patterns of use and experience of the website, and how the toolkit could be improved. The framework evolved during familiarisation and indexing to incorporate additional issues raised by participants.

## Results

**Fig. 1:**
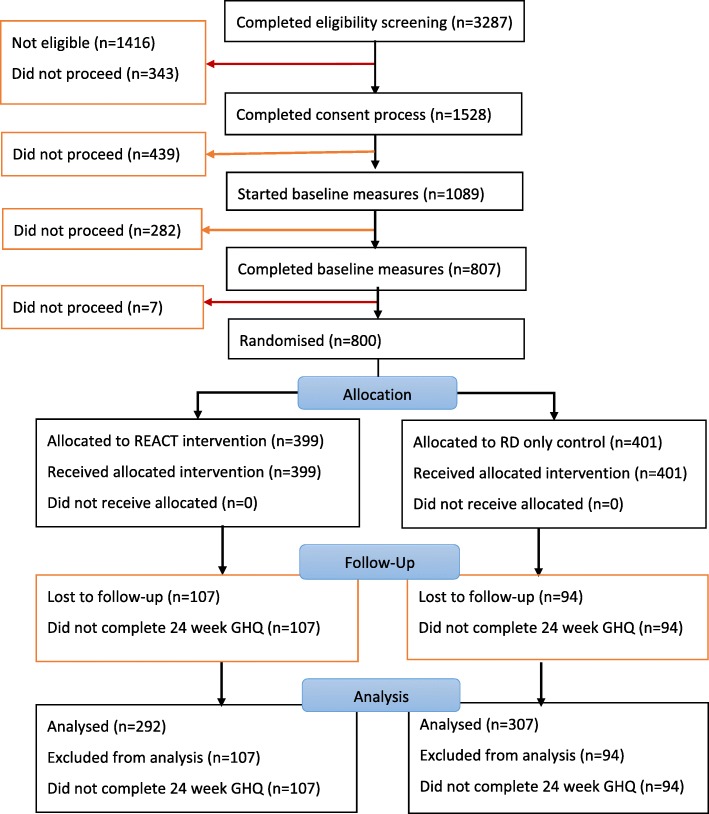
REACT flow diagram

The flow diagram (Fig. 1) shows recruitment and retention throughout the study. There were 4348 registration page visits. Of 3287 people who completed eligibility screening, 1416 failed on at least one criteria, with 1146 (81%) of these failing to report higher than usual levels of distress. Of the 1528 (46%) who subsequently provided consent for the study, 807 completed baseline measures and 800 (52% of those consenting) were randomised. Unfortunately due to an administrative error, detailed web usage data was not collected from the outset and so was only available for 700 of the 800 participants (REACT = 348; RD only = 352). There were nine instances of unblinding in total.

Baseline demographic and situational characteristics of the participants are presented in Table 1.

**Table 1:** Demographic and situational characteristics of participants

	REACT *n* = 399	RD *n* = 401	Overall *n* = 800
**Age (years)**			
< 30	39 (9.77)	36 (8.98)	75 (9.38)
30–39	50 (12.53)	73 (18.20)	123 (15.38)
40–49	95 (23.81)	104 (25.94)	199 (24.88)
50–59	111 (27.82)	112 (27.93)	223 (27.88)
60–69	88 (22.06)	61 (15.21)	149 (18.63)
≥ 70	16 (4.01)	15 (3.74)	31 (3.88)
Mean (SD)	49.4 (13.3)	47.9 (12.7)	48.6 (13.00)
Range (min–max)	16–84	18–86	16–86
**Gender**			
Male	82 (20.55)	69 (17.21)	151 (18.88)
Female	317 (79.45)	331 (82.54)	648 (81.00)
Missing	0 (0.00)	1 (0.25)	1 (0.13)
**How many people do you support?**			
1	296 (74.19)	295 (73.57)	591 (73.88)
2	68 (17.04)	72 (17.96)	140 (17.50)
3	20 (5.01)	21 (5.24)	41 (5.13)
≥ 4	15 (3.76)	13 (3.24)	28 (3.50)
**Relationship to service user**			
Mother	187	200	387
Father	17	10	27
Partner	149	143	292
Daughter	56	62	118
Son	6	1	7
Sibling	41	38	79
Friend	31	26	57
Grandparent	8	2	10
Wider family member	17	17	34
Other	10	12	22
Undefined	38	52	90
**Ethnicity**			
White British	361 (90.48)	366 (91.27)	727 (90.88)
White Irish	5 (1.25)	6 (1.50)	11 (1.38)
Any other white background	15 (3.76)	13 (3.24)	28 (3.50)
Mixed	6 (1.50)	6 (1.50)	12 (1.50)
Asian or Asian British	11 (2.76)	3 (0.75)	14 (1.75)
Other Ethnic group	1 (0.25)	5 (1.25)	6 (0.75)
Rather not say	0 (0.00)	2 (0.50)	2 (0.25)
**Marital status**			
Single	88 (22.06)	77 (19.20)	165 (20.63)
Married	219 (54.89)	239 (59.60)	458 (57.25)
Civil partnership	14 (3.51)	13 (3.24)	27 (3.38)
Separated	8 (2.01)	15 (3.74)	23 (2.88)
Divorced	47 (11.78)	40 (9.98)	87 (10.88)
Widowed	10 (2.51)	8 (2.00)	18 (2.25)
Rather not say	13 (3.26)	9 (2.24)	22 (2.75)
**Living arrangements**			
Spouse or partner	275 (68.92)	289 (72.07)	564 (70.50)
Living alone	82 (20.55)	80 (19.95)	162 (20.25)
Parent(s)	17 (4.26)	11 (2.74)	28 (3.50)
Other	20 (5.01)	17 (4.24)	37 (4.63
Rather not say	5 (1.25)	4 (1.00)	9 (1.13)
**Dependents**			
None	168 (41.90)	175 (43.86)	343 (42.88)
1	99 (24.69)	117 (29.32)	216 (27.00)
2	91 (22.69)	57 (14.29)	148 (18.50)
3	30 (7.48)	28 (7.02)	58 (7.25)
≥ 4	13 (3.26)	22 (5.49)	35 (3.48)
**Highest education level**			
School level	65 (16.29)	73 (18.20)	138 (17.25)
Further education (college)	108 (27.07)	117 (29.18)	225 (28.13)
Higher (University)	226 (56.64)	211 (52.62)	437 (54.63)
**Employment status**			
Employed full-time (35 h + a week)	150 (37.59)	151 (37.66)	301 (37.63)
Employed part-time	92 (23.06)	96 (23.94)	188 (23.50)
Unable to work due to caring responsibilities	33 (8.27)	33 (8.23)	66 (8.25)
Unable to work due to ill health/disability	30 (7.52)	20 (4.99)	50 (6.25)
Unemployed	10 (2.51)	8 (2.00)	18 (2.25)
Student	7 (1.75)	8 (2.00)	15 (1.88)
Retired	53 (13.28)	58 (14.46)	111 (13.88)
Voluntary work	12 (3.01)	11 (2.74)	23 (2.88)
Housewife/house husband	12 (3.01)	16 (3.99)	28 (3.50)
**Home internet access**			
Yes	395 (99.00)	400 (99.75)	795 (99.38)
No	1 (0.25)	0 (0.00)	1 (0.13)
Intermittent or poor quality	3 (0.75)	1 (0.25)	4 (0.50)
**Paid work affected by caring role**			
No, I didn’t have paid work before	120 (30.08)	125 (31.17)	245 (30.63)
No, I still perform the same amount of paid work	198 (49.62)	195 (48.63)	393 (49.13)
Yes, I stopped work completely	40 (10.03)	33 (8.23)	73 (9.13)
Yes, I reduced my working hours	41 (10.28)	48 (11.97)	89 (11.13)
Mean (SD)	13.5 (9.3)	11.4 (6.6)	12.4 (8.0)
Min–max	2–48	1–30	1–48

Participants were typically middle aged (53% aged 40–60), white British (91%), female (81%), mothers (48%), and highly educated (55% to university level). The majority were supporting young adults aged 35 or less (61%), more than half of whom (58%) had a diagnosis of bipolar. Most were supporting only one person with a mental health problem, but 26% reported supporting two or more people, and 57% had other dependents. Some 61% were married or in a civil partnership. Most were in full-time, part-time or voluntary work (64%) but 8.5% reported being unable to work specifically due to their caring responsibilities. All except four participants had home internet access.

Baseline, 12 and 24 week scores are presented in Table 2. Relatives reported very high levels of distress at baseline (mainly due to the inclusion criteria on GHQ-28 score). 784 of the 800 (89%) scored at or above 24, generally considered to be a screening threshold for psychiatric caseness [49, 50]. Highest scores were on the anxiety/insomnia subscale.

**Table 2:** Baseline, 12 and 24 week scores: values are mean (SD) unless stated otherwise

	REACT	RD	Total
GHQ-28			
Baseline	40.3 (14.6)	40.0 (14.0)	40.2 (14.3)
12 weeks	30.6 (15.2)	32.9 (15.4)	31.8 (15.3)
24 weeks	29.6 (15.9)	31.3 (15.2)	30.5 (15.6)
GHQ-28 subscales			
Somatic symptoms			
Baseline	10.3 (4.4)	10.4 (4.0)	10.3 (4.2)
12 weeks	8.1 (4.3)	8.7 (4.4)	8.4 (4.4)
24 weeks	7.9 (4.7)	8.3 (4.5)	8.1 (4.6)
Anxiety/insomnia			
Baseline	13.0 (4.1)	12.9 (4.0)	13.0 (4.1)
12 weeks	9.5 (4.7)	10.1 (4.8)	9.8 (4.7)
24 weeks	9.2 (4.9)	9.9 (4.9)	9.6 (4.9)
Social dysfunction: note that values are median (IQR)			
Baseline	11 (8–13)	11 (8–14)	11 (8–13.5)
12 weeks	8 (7–11)	9 (7–13)	9 (7–12)
24 weeks	8 (7–11)	8 (7–11)	8 (7–11)
Severe depression: note that values are median (IQR)			
Baseline	4 (1–9)	4 (1–9)	4 (1–9)
12 weeks	2 (0–7)	3 (0–7)	2 (0–7)
24 weeks	2 (0–6)	2 (0–7)	2 (0–7)
CWS - wellbeing			
Baseline	55.9 (25.9)	55.8 (26.4)	55.9 (26.1)
12 weeks	72.0 (27.0)	68.9 (27.7)	70.3 (27.4)
24 weeks	77.0 (26.6)	72.6 (30.5)	74.7 (28.8)
CWS - support			
Baseline	19.5 (11.6)	18.8 (11.7)	19.1 (11.7)
12 weeks	26.0 (12.0)	22.6 (12.0)	24.2 (12.1)
24 weeks	25.7 (11.7)	23.2 (12.2)	24.4 (12.0)

Mean wellbeing scores on CWS were in the 50s at baseline (possible range is 0–128, higher scores indicate greater wellbeing). Mean support scores at baseline were below 20 in each group (possible range 0–51, higher scores indicate greater support). Although there are no clinical thresholds for CWS, wellbeing and support scores were very low compared to other studies of groups of relatives of people with psychosis [6, 51, 52].

Taking into account full costs of development and delivery (shown in Table 3), REACT cost £142.95 per person, and RD only £0.84. Most of these costs were development; ongoing delivery would cost £62.27 for REACT and £0.43 for RD.

**Table 3:** Development costs for REACT and RD

REACT Development Type of cost	Total no. of hours or units	Cost per hour or unit	Total
Content generation			£5574.40
Staff			£3699.40
Professor of clinical psychology	54 h	£55.81	£3013.74
Clinical psychologist	17 h	£29.66	£504.22
Research assistant	18 h	£10.08	£181.44
Relatives			£1875
Relative co-applicant	29 h	£20	£580
Relatives in focus groups and advisory role	118 h	£10	£1180
Relatives’ travelling	23 persons	£5	£115
Producing videos and images			£18,422.78
Staff			£14,326.13
Research fellow	450 h	£23.76	£10,692
Research assistant	157.5 h	£15.83	£2493
Information officer	37.5 h	£30.43	£1141.13
Communications and information manager	56.25 h	£31.36	£1764
Actors			£2112.65
Relatives	11 persons	£20/person	£220
Developing and designing the website			£12,499.59
Staff			£10,901.11
Professor of clinical psychology	36 h	£55.81	£2009.16
Professor of clinical psychology	26 h	£69.80	£1814.80
Professor of psychiatry	26 h	£68.00	£1768.00
Research assistant	10 h	£12.29	£122.90
Digital technologist/web developer	225 h	£23.05	£5186.25
Relatives			£1598.48
Relatives’ focus groups	56 h	£20	£1120.00
Other relatives	8 h	£59.81	£478.48
Website infrastructure during development (until going live)			£28,039
Domain name			£9
SSL certificate fees			£30
Web hosting and exclusive IP address			£100
Website development			£27,900
Total			£64,535.99
REACT Delivery Type of costs	Total no. of hours or units	Cost per hour or unit	Total
General infrastructure for hosting REACT			£5119
Digital technology/web developer	180 h	£23.05	£4149
Secure web hosting and exclusive IP address	6 months	£100	£600
Software for bulk emails	2 blocks	£185	£370
Training, supervision and employment (6 months) of REACT supporters			£20,813.05
REACT supporters	756 h	£15.83	£11,967.48
Back-up REACT supporter	94 h	£13.52	£1270.88
Expert relative REACT supporter	47 h	£20	£940
Supervision	33 h	£64.71	£2135.52
In-house training	224.75 h	£18.33	£4119.17
External training			£380
Recruitment			£12,635.56
Adverts (Facebook, Google and Bipolar UK)			£11,059.56
Printing			£1526.00
Flyers and postage			£50.00
Total			£38,567.61
Resource Directory Development Type of costs	Total no. of hours or units	Cost per hour or unit	Total
Development costs			£463.50
Staff			£324.50
Research assistant	20 h	£10.08	£201.60
Research assistant	10 h	£12.29	£122.90
Infrastructure			£139.00
Domain name	1	£9.00	£9.00
SSL certificate fees	1	£30.00	£30.00
Web hosting and exclusive IP address	1	£100.00	£100.00
Resource Directory Delivery costs			£205.79
REACT supporter	13 h	£15.83	£205.79
Total			£669.29

*Average costs were calculated as follows:*

*• Total development costs: 800 users (total trial participants)*

*• General website infrastructure costs: 800 users (total trial participants)*

*• REACT supporter costs: 400 users (participants in REACT trial arm)*

*• Recruitment costs: 3287 users (number that initiated a registration in the REACT website)*

The median time spent online on REACT was 50.8 min (IQR 12.4–172.1) compared to 0.5 min (IQR 0–1.6) on RD only. REACT was accessed more outside traditional working hours (9 am–5 pm Monday to Friday excluding public holidays) (median 33.6 min (IQR 7.2–10.2)) than during (median 24.5 min (IQR 4.8–64.9)). The most popular module was the online forum (60% REACT participants visited at least once). However, of the 207 visitors, only 67 were actively posting, with a mean number of 9.8 (SD 25.9) posts each. The least popular were “Recovery: looking to the future” and “Managing stress; thinking differently” (31% visited). Detailed descriptions of REACT module levels of use are shown in Table 4.

**Table 4:** REACT module usage- time is reported in minutes. Data is based on *n* = 348

**MODULE 1 – What is psychosis**	
**Total time spent on page per person (mins)**	
Number of people who accessed page	205
Total time (across all participants)	188.4
Mean time on page per person (STD)	11.4 (13.1)
Median time on page per person (IQR)	1.4 (0.5, 5.5)
Min – max time spent on page	0, 55.8
**MODULE 2 – What is bipolar disorder**	
**Total time spent on page per person (mins)**	
Number of people who accessed page	203
Total time (across all participants)	158.7
Mean time on page per person (STD)	14.6 (17.4)
Median time on page per person (IQR)	8.2 (2.3, 20.4)
Min – max time spent on page	0.1, 97.4
**MODULE 3 – Managing positive symptoms**	
**Total time spent on page per person (mins)**	
Number of people who accessed page	163
Total time (across all participants)	167.1
Mean time on page per person (STD)	13.1 (15.6)
Median time on page per person (IQR)	5.7 (1.8, 20.4)
Min – max time spent on page	0, 75.8
**MODULE 4 – Managing negative symptoms**	
**Total time spent on page per person (mins)**	
Number of people who accessed page	153
Total time (across all participants)	127.5
Mean time on page per person (STD)	14.2 (23.2)
Median time on page per person (IQR)	4.6 (1.2 18.1)
Min – max time spent on page	0.1, 167.3
**MODULE 5 – Managing mood swings**	
**Total time spent on page per person (mins)**	
Number of people who accessed page	134
Total time (across all participants)	64.3
Mean time on page per person (STD)	7.3 (10.4)
Median time on page per person (IQR)	3.4 (0.6, 8.6)
Min – max time spent on page	0.1, 59.1
**MODULE 6 – Dealing with difficult situations**	
**Total time spent on page per person (mins)**	
Number of people who accessed page	145
Total time (across all participants)	117.6
Mean time on page per person (STD)	11.8 (14.5)
Median time on page per person (IQR)	6.3 (1.6, 16.3)
Min – max time spent on page	0.1, 75.3
**MODULE 7 – Managing stress – doing this differently**	
**Total time spent on page per person (mins)**	
Number of people who accessed page	133
Total time (across all participants)	75.2
Mean time on page per person (STD)	14.5 (25.3)
Median time on page per person (IQR)	5 (1.2, 17.4)
Min – max time spent on page	0, 194.8
**MODULE 8 – Managing stress – thinking differently**	
**Total time spent on page per person (mins)**	
Number of people who accessed page	108
Total time (across all participants)	52.2
Mean time on page per person (STD)	6.5 (7.8)
Median time on page per person (IQR)	3.4 (1.2, 9.3)
Min – max time spent on page	0, 37.5
**MODULE 9 – Understanding mental health services**	
**Total time spent on page per person (mins)**	
Number of people who accessed page	126
Total time (across all participants)	78.9
Mean time on page per person (STD)	11.7 (22.6)
Median time on page per person (IQR)	3.9 (0.4, 14.4)
Min – max time spent on page	0, 136
**MODULE 10 – Treatment options**	
**Total time spent on page per person (mins)**	
Number of people who accessed page	140
Total time (across all participants)	152.3
Mean time on page per person (STD)	12.5 (30.0)
Median time on page per person (IQR)	5.1 (1.2, 14.7)
Min – max time spent on page	0, 329.7
**MODULE 11 – Dealing with crises**	
**Total time spent on page per person (mins)**	
Number of people who accessed page	113
Total time (across all participants)	66.5
Mean time on page per person (STD)	9.2 (16.9)
Median time on page per person (IQR)	3.3 (0.8, 9.2)
Min – max time spent on page	0, 129.3
**MODULE 12 – Recovery: looking to the future**	
**Total time spent on page per person (mins)**	
Number of people who accessed page	108
Total time (across all participants)	97.6
Mean time on page per person (STD)	10.1 (13.8)
Median time on page per person (IQR)	4.4 (1.3, 15.1)
Min – max time spent on page	0, 77.7
**FORUM**	
**Total time spent on page per person (mins)**	
Number of people who accessed page	207
Total time (across all participants)	209.3
Mean time on page per person (STD)	65.0 (201.2)
Median time on page per person (IQR)	12.1 (2.2, 58.4)
Min – max time spent on page	0, 2553.8
**DIRECT MESSAGING**	
**Total time spent on page per person (mins)**	
Number of people who accessed page	141
Total time (across all participants)	72.3
Mean time on page per person (STD)	15.8 (40.1)
Median time on page per person (IQR)	0.7 (0.2, 7.2)
Min – max time spent on page	0, 260.7

Retention to the primary outcome was 74% at 12 weeks, and 75% at 24 weeks (REACT *n* = 292 (73.2%); RD-only *n* = 307 (76.6%)). Similar numbers of participants dropped out in each arm, but those who dropped out in the REACT arm were more distressed than those who remained. The joint model estimates that the REACT arm participants who dropped out were on average 0.33 (95% CI -0.27–0.93, *p* = 0.279) GHQ units more distressed than those who did not drop out; note however that this is the average over the entire 24-week period, i.e. this model assumes that the difference in distress between those REACT participants who did and did not drop out was constant over the entire period. In the RD only arm, the equivalent result for those who did/not drop out = 0.12 (95% CI -0.52–0.77, *p* = 0.707). This meant that data could not be assumed to be missing at random.

Relatives’ distress decreased significantly in both groups by 24 weeks (GHQ-28; average daily reduction = −.06, 95% CI = − 0.06, − 0.05, *p* < 0.001). The estimated mean difference between the two groups on the primary outcome at 24 weeks favoured REACT but was small (− 1.39, 95% CI = − 3.60, 0.83) and not statistically significant (*p* = 0.2189). At 12 weeks’ follow-up, GHQ-28 scores were lower in REACT than in RD (− 2.08, 95% CI = − 4.14, − 0.03), and although statistically significant (*p* = 0.027), this was likely to be of limited clinical significance. After accounting for missing data in a longitudinal model, there was no significant difference between the REACT and RD arms over the 24-week follow-up period (− 0.56, 95% CI = − 2.34, 1.22, *p* = 0.51).

When adjusting for baseline GHQ-28, being male, single, and unemployed (or in unpaid work) were all significantly associated with greater levels of distress at 24 weeks (Table 5).

**Table 5:** Multivariable analyses, adjusting for baseline GHQ-28 and significant baseline covariates (stepwise selection)

Covariate	Coefficient (95% CI)	*p*-value
Treatment	−1.48 (−3.80, 0.85)	0.2121
Baseline GHQ-28	0.51 (0.42, 0.59)	<.0001
Gender (Male vs. reference category: Female)	3.39 (0.27, 6.51)	0.0334
Marital status (Married/civil partnership vs. reference category: Single/divorced/separated/widowed)	−3.65 (−6.11, − 1.18)	0.0038
Employment (reference category: None/unpaid)		0.0039
Part-time	−2.10 (−5.11, 0.91)	
Full-time	−4.60 (−7.30, −1.90)	

Number included in analysis - REACT: *N* = 292; RD: *N* = 307

Carer wellbeing and support both increased significantly over time in both groups (wellbeing = 0.11, 95% CI 0.09, 0.12, *p* < 0.001; support = 0.03, 95% CI 0.02, 0.03, *p* < 0.001). There were no significant differences between groups in wellbeing at either 12 weeks (1.53, 95% CI = − 2.21, 5.27, *p* = 0.42) or 24 weeks (2.39, 95% CI = − 1.76, 6.54, *p* = 0.26). Relatives in REACT reported higher levels of support at 12 weeks (2.50, 95% CI = 0.87, 4.12, *p* < 0.0001) and at 24 weeks (1.65, 95% CI = 0.04, 3.27, *p* = 0.045). However, after accounting for missing data in a longitudinal model, the mean difference (1.51, 95% CI -0.005, 3.01) was no longer statistically significant (*p* = 0.051) and was unlikely to be of clinical significance.

The mean number of web page downloads in the REACT group was 149.9 (SD 266). For each additional download, there was an average reduction of 0.01 in GHQ-28 at 24 weeks; however, this effect was not statistically significant (− 0.01, 95% CI = − 0.02, 0.01, *p* = 0.30).

Participants reported finding REACT a safe and confidential environment (96%), feeling supported by the REACT group (89%) and REACT supporters (86%). There were no high risk adverse events or reported side effects.

Twenty four of fifty-five participants who were invited to take part in a qualitative interview did so; 10 declined, 10 did not respond and 11 were unavailable within the narrow time period available for this data collection. Consistent with the whole trial sample, participants were typically middle aged (median age 54 yrs. (range 26–69); predominantly female (*n* = 20, 83%) and white British (*n* = 21, 88%). Qualitative feedback (indicative quotes shown) was extremely positive. REACT was particularly valued by relatives for being comprehensive, relevant, easy to access, private and anonymous.

Mother, 54: *The great thing is that you can just go when you need to, as opposed to having to make appointments and get to a place*.

Mother, 57: *You are anonymous. And you can leave when you want, whereas if you go to a group you tend to be there for at least a polite amount of time.*

Male partner, 35: *The layout was quite easy to use and stuff like that, it broke down everything that you need from, you know, different kinds of mental illness – my wife was diagnosed with bipolar so … while I knew about depression or I had a little understanding of depression, bipolar I didn’t know at all. And it was quite good to … get an eye-opener on different treatments and it was the first place I went to when my wife went through ECT treatment as well, because it kind of helped me cope with it.*

The proactive support from REACT supporters was appreciated, as was the opportunity to learn through a variety of different media (text, video, forum).

Mother, 65: *So I got the responses from the REACT supporters about … how to navigate the system differently and language to use and so on and so forth, and then [from] people who had had … much worse experiences than my experience. So it was that ability to connect with people who kind of have some empathy with what’s going on in your life and how difficult it can be in those moments.*

Sister, 26: *The videos were really helpful because it wasn’t constant reading so I like that. I loved the depth of information that was available. The layout itself was absolutely great as well, it was easy to read, it was eye-catching enough and quite interactive as well … The opportunity for me to be able to write notes and things like that, I thought that was really, really good.*

A consistent message was that REACT would be most useful to relatives early in the recovery journey, when they were likely to be seeking information and strategies.

Female partner, 43: *We only had a diagnosis last year, so actually I was really desperate for any resources and any further information that I could find, so I was literally soaking everything up as much as I could, and I found REACT through Bipolar UK and … it has been really helpful because I think what I really struggled to find was anybody else in a similar situation who had a recent diagnosis, you know early forties and [with] a young family*

Some relatives found seeking help for their own needs difficult, and most relatives found prioritising time to use REACT difficult.

Mother, 57: *I was engaging with it [REACT] and then he then went into crisis and then went into hospital and in fact he was in hospital until the following January, and I was then caught up in that. And then you know after that I needed reminders. So I think that’s your difficulty really, is that the very people that you’re trying to help have so much on their plates really.*

## Discussion

In the first randomised controlled trial of a digital intervention to support relatives of people with psychosis or bipolar, the Relatives Education And Coping Toolkit (REACT), including 12 psychoeducation modules, a moderated online forum, confidential direct messaging service, and a comprehensive Resource Directory was compared to the Resource Directory only. Relatives reported high levels of distress (GHQ-28 primary outcome) at baseline, which reduced significantly in both groups over the 24 weeks follow-up period, but there was no difference between the groups at follow up. Carer wellbeing and support scores (CWS) were very low at baseline and increased significantly in both groups, with no significant differences between groups. Changes over time may reflect regression to the mean. There were no adverse events: relatives using REACT reported feeling safe and supported, and qualitative experiences of using REACT were positive.

REACT offers an inexpensive, safe and acceptable way to deliver NICE recommended information and support to relatives of people with severe mental health problems, but there was no evidence that it reduces distress more effectively than a comprehensive resource directory. These findings are consistent with previous studies showing that in general, interventions designed to improve outcomes for relatives are less effective for those with higher levels of distress [26], This may be due to the impact of other life challenges that cause distress and therefore impact on GHQ scores, but are unrelated to the caring role, including being male, single, and not in paid work, which were significant predictors of the primary outcome but which are unlikely to be addressed by carer interventions such as REACT. Targeting relatives with lower levels of generic distress or using a more specific measure of distress associated with caring may have led to different outcomes. Another possible explanation for lack of a significant clinical effect of REACT is low levels of website use compared to carer support delivered face-to-face. This pattern of use is consistent across digital health interventions and may paradoxically stem from their inherent flexibility of digital interventions [53]. REACT was accessible at any time, and relatives were given no expectations of times, levels, or order of use. The REACT Supporters proactively engaged with activity on the forum and direct messages, but use of the psychoeducational modules was unsupported. Clearer expectations of use and feeling accountable to a supporter may have enhanced engagement.

### Strengths and limitations

This trial was rigorously conducted, with a large, broadly recruited sample, clearly defined and theoretically based supported intervention, an active control group, good follow-up rate for an online trial, web-based randomisation, robust blinding, and a pre-published analysis plan that appropriately addressed missing data. The key limitations were: failure to recruit more men and people from ethnic monitory groups, which limits the generalisability of the findings; and (with hindsight) the inclusion of GHQ-28 minimal score as an inclusion criterion, which limited the sample to highly distressed relatives, increasing the likelihood of regression to the mean in both arms of the study over the follow-up period. Non-random dropout (greater in participants with higher baseline GHQ-28 scores) further limited the potential to identify group differences, though this was robustly dealt with using a joint modelling approach.

### Implications for future research

The findings highlight two key lessons for research in digital health interventions. The first is that we cannot assume that online interventions adapted from those delivered face-to-face will be equally effective: REACT draws on evidence based cognitive behavioural interventions [34], and was shown to be effective in reducing distress when offered in paper form and supported by telephone by staff linked to the relevant clinical team [6]. It is not possible to determine in this study whether the lack of an effect is due to delivering REACT online, supporting REACT online with trained relatives, or the higher levels of relatives’ distress at baseline.

The second is that we need new methodologies appropriate to the rigorous evaluation of digital health interventions. They must be controlled: without an active control group, a pre-post evaluation of REACT would have made it appear very effective. They must account for higher levels of dropout and missing data: without accounting for non-random missing data, REACT would have appeared a more effective intervention than RD-only in improving relatives’ support. However, they also need to allow a more flexible iterative development of the technology in response to feedback, to test the technology as one component of a much broader care package within context, and to establish which part of an intervention has what effect on which people. In this study, REACT remained relatively fixed throughout the trial (excluding updating directory for accuracy), despite ongoing feedback about ways it could have been improved, and general advances in website design. We also do not understand exactly what relatives did in response to using REACT or the RD. In particular, whether or not they sought support from organisations recommended in the RD or how effective this was. Alternative methodologies such as iterative testing and adaptation suggested by Mohr et al., [54] or those based on realist approaches [55] may offer useful ways forward.

## Conclusions

Relatives need access to information and emotional support. REACT offers an inexpensive, safe and acceptable way to deliver this, even if it does not reduce their distress. Therefore, REACT should continue to be developed in light of user feedback, and offered and evaluated as one component of a comprehensive care package, which includes face-to-face support.

## Data Availability

The CTRC trial statisticians had access to the data throughout the trial. A de-identified version of the dataset will be transferred to the sponsor (Lancaster University) by March 31 2020. Ownership of copyright and intellectual property rights for all research conducted for the REACT study will ultimately be held by Lancaster University. We intend to make available individual participant data that underlie the results reported in this article, after de-identification. Data will be made available on request 12 months following article publication, and only to researchers who provide a methodologically sound proposal and where the proposed use of the data has been approved by an independent ethics review committee (“learned intermediary”) identified for this purpose. Proposals should be directed to rdm@lancaster.ac.uk. Data will be available for 10 years at Lancaster University’s Research Directory (10.17635/lancaster/researchdata/306).

## Abbreviations

REACT: Relatives’ Education And Coping Toolkit
RD: Resource Directory
GHQ: General Health Questionnaire
NICE: National Institute for Health and Clinical Excellence
NHS: National Health Service
TM: Trial Manager
CWS: Carer Wellbeing & Support scale
